# Spontaneous Rectus Sheath Hematoma in the Elderly: An Unusual Case and Update on Proper Management

**DOI:** 10.1155/2014/675678

**Published:** 2014-03-11

**Authors:** George Galyfos, Georgios Karantzikos, Konstantinos Palogos, Argiri Sianou, Konstantinos Filis, Nikolaos Kavouras

**Affiliations:** ^1^Department of General Surgery, General Hospital of Chalkis, 34100 Chalkis, Greece; ^2^Department of Microbiology, General Hospital of Chalkis, 34100 Chalkis, Greece; ^3^1st Department of Propaedeutic Surgery, University of Athens Medical School, Ippokration Hospital, Athens, Greece

## Abstract

Spontaneous rectus sheath hematoma (SRSH) is an uncommon medical emergency in the elderly. We present a case of SRSH with an atypical clinical presentation and discuss literature regarding diagnosis and proper management. A 75-year-old female patient was transferred to the emergency department due to acute dyspnoea and confusion. Her medical history revealed a viral infection of the upper respiratory tract, and no coughing or use of anticoagulants. The clinical examination showed tenderness of the left lower abdomen, although palpation was misleading due to patient's obesity. Laboratory investigations showed light anaemia. Ultrasonography and computed tomography revealed a large rectus sheath hematoma of the left abdominal wall. Despite further deterioration of the patient, conservative management including bed rest, fluid replacement, blood products transfusion, and proper analgesia was successful. No surgical intervention was needed. Prompt diagnosis and management of SRSH plays significant role in the prognosis, especially in elder patients. Independently of size and severity, conservative management remains the first therapeutic choice. Only by failure of supportive management, progressive and large hematoma or uncontrollable hemodynamic patients, interventional management including surgery or less invasive newer techniques is indicated.

## 1. Introduction

Spontaneous rectus sheath hematoma (SRSH) is an uncommon entity [1]. The aging population and increased use of anticoagulant therapy without adequate control of coagulation parameters have caused a significant rise in the incidence of this condition in the last decades [2, 3]. SRSH is usually self-limiting but can evolve to a life-threatening emergency, especially for the elderly [2, 4]. This is mainly due to the low health status and various comorbidities that are observed in this group of patients, whereas delayed diagnosis plays a significant role as well.

We present a case of an elder patient with SRSH and an unusual clinical presentation. We highlight the importance of prompt diagnosis and management of this medical emergency and include a review of the literature.

## 2. Case Report

A 75-year-old woman was transferred to our emergency department due to sudden onset of dyspnoea and confusion. She reported nasal congestion, sneezing, and malaise during the last three days, indicating a possible viral infection. The patient reported no significant comorbidities except from arterial hypertension under therapy, no coughing, and no anticoagulant use.

On examination, her temperature was 37.0°C, blood pressure 110/85 mmHg, pulse rate 100 beats/min, and respiratory rate over 25 breaths/min. The auscultation of the thorax did not reveal any abnormal respiratory or cardiac sounds. The abdominal examination revealed sensitivity in the lower left abdomen with active bowel sounds. Due to patient's obesity (BMI = 39.1), no mass was palpable during examination. Guarding was felt mainly on the same side, whereas the right side was not tender. The remaining physical examination was unremarkable. The patient was examined by a surgeon as well.

Laboratory findings revealed a light leukocytosis (11000/mm^3^), a light anaemia (33.5%), and normal platelet number (300000/mm^3^). The rest of laboratory studies were as follows: ALT 25 U/L, AST 32 U/L, glucose 120 mg/dL, alkaline phosphatase 225 U/L, total bilirubin 1.3 mg/dL with a direct fraction of 0.5 mg/dL, creatinine 1.3 mg/dL, and albumin 6/mg/dL. Electrolytes, analysis of urine, bleeding and clotting time, chest X-ray, and electrocardiogram were normal. An abdominal ultrasonography study was ordered demonstrating a left sided rectus sheath hematoma measuring 10 cm × 10 cm. Computed tomography confirmed these findings.

During her stay in the emergency department, the patient presented a decrease in her blood pressure (90/70 mmHg) and further deterioration. Resuscitation with fluid replacement and adequate analgesia followed. Diuresis was not decreased significantly. The patient became stable and remained in the hospital for monitoring. During her hospital stay, the patient showed a further decrease in her hematocrit levels which improved after transfusion of blood products. Overall, no surgical intervention was needed.

## 3. Discussion

SRSH is more common in elderly women than in men, the proportion being 2-3/1 [1, 2]. This could be explained as the protection provided by the anatomy of the rectus sheath may be compromised by decreased muscle and age-related changes from arteriosclerosis, or hypertension that may render vessels more susceptible to injury [4]. Although the etiology includes trauma, abdominal operations, trocar site injury after laparoscopic operations, subcutaneous drug injections, anticoagulant therapy, hematological diseases, coughing, physical exercise, and pregnancy, it rarely occurs spontaneously [5, 6]. In our case, the patient's history did not reveal any other probable cause, except for sneezing.

Regarding diagnosis, our case presented with an unusual clinical picture that misguided the diagnosis. Common presenting signs and symptoms of SRSH are abdominal pain, palpable abdominal wall mass, abdominal wall ecchymosis, nausea, vomiting, tachycardia, peritoneal irritation, fever, abdominal distention, and abdominal cramping [7, 8]. Fothergill's sign and Carnett sign are usually positive in rectus sheath hematoma and help to differentiate this condition from intra-abdominal pathologies, although our patient's obesity made the identification of the aforementioned signs difficult [9]. Fothergill's sign is positive when the hematoma within the rectus sheath produces a mass that does not cross the midline and remains palpable when the patient tenses his rectus muscle by touching his chest using his chin. Carnett sign is the exacerbation of pain and tenderness over the hematoma by contraction of rectus muscle by sitting halfway up in a supine position. The presentation of SRSH is indeed more likely to be atypical in elderly persons [2, 4]. Abdominal pain may not be present. SRSH has been reported in elderly patients with chief symptoms of distress or urinary retention [8, 9].

Our case confirms that laboratory findings may demonstrate a decrease in the haemoglobin level, although this may be misleading early in the course. Leukocytosis, thrombocytosis, and prolonged clotting studies in patients on oral anticoagulation may also be present [9]. Patients on warfarin must be under close monitoring of INR. Patients with INR level >3 have shown a 5 times increased risk of hemorrhagic complication [10]. In addition, hypertension and renal or cerebrovascular diseases show a high prevalence in the elderly and significantly increase the bleeding risk [11]. Moreover, the high incidence of dementia in the elderly leads sometimes to overdosage of oral anticoagulants, increasing by this way the risk for SRSH [4, 11].

Both ultrasound examination (US) and computed tomography (CT) are the indicated diagnostic modalities; in our case, they identified the hematoma without doubt [12, 13]. Both methods are useful for differentiating intra-abdominal pathologies and reducing the possibility for unnecessary laparotomy [14]. Ultrasonography remains the first choice as it shows cost and time effectiveness, and it equals with less radiation for the patient, although its sensitivity ranges from 70% to 90% in published reviews [1, 12]. However, CT has 100% sensitivity and specificity for SRSH; therefore, it remains useful for confirming the diagnosis, because most of these patients are elderly cardiac patients, and several acute abdominal conditions including mesenteric ischemia, rupture of an abdominal aneurysm, peptic ulcer disease, and perforation secondary to aspirin must be excluded [4, 5, 14]. CT can also identify whether the bleeding is active or not. Moreover, CT appearance can be useful to classify SRSH into three subtypes based on size and severity (types I–III) [13]. Large hematomas, such as in our case, are considered to be of types II and III.

The difficulties in the correct diagnosis frequently lead to delay in treatment or unneeded surgery. Our patient corresponded to conservative management, as most cases do [15, 16]. Reversal of anticoagulation and resuscitation with fluids and blood products are necessary in general, but anticoagulation is crucial in patients with prosthetic valves, as they have acquired thrombotic diathesis [17]. In Type I hematomas, hospitalisation is not usually required, and the hematoma resorbs spontaneously within 30 days. In Type II lesions, bed rest, intravenous fluid replacement, and analgesia are the appropriate treatment. In Type III lesions, additional blood product transfusions are required. <6> In type II and III hematomas, normalization of coagulation parameters by administration of vitamin K1 and fresh frozen plasma may also be needed [15, 16]. Hence, the need for anticoagulation should be weighed against the risk of rebleeding once the patient is stabilized. In the study by Cherry and Mueller, only 4.8% of the patients had a repeat episode of RSH after the anticoagulation therapy was restarted [8]. Reinitiating anticoagulation was shown to be safe in high-risk patients for thromboembolic events [16–18].

Clinical improvement is usually rapid in patients with type I or II SRSH, although our patient was fully stabilized after a week. Spontaneous complete resolution, especially in large hematomas, may take up to three months [19]. In hemodynamically stable patients, the common management, according to some studies, currently continues to be conservative by suspension of the anticoagulation treatment, correction of the anticoagulation state, volume resuscitation, and supportive measures, especially for the elderly [16, 18, 20].

Surgical intervention includes evacuation of the hematoma and ligation of the offending epigastric vessel. Surgery is indicated, by most authors, only for cases that do not respond to supportive management, progressive large hematoma, or uncontrollable hemodynamic patients [15, 20]. However, there are reports where conservative management is adequate and effective for large hematomas as well. In the Berná et al. study, the authors conclude that with early diagnosis and conservative management surgical intervention can be avoided even with large hematomas [16]. Alla et al. underline that invasive procedures or surgery are rarely needed for securing hemostasis and stabilizing hemodynamics [21]. Surgical management is associated with significant morbidity due to the advanced age and multiple comorbidities in elder patients [20]. Therefore, it is reserved for the most severe cases.

Regarding alternative interventional options, new methods have become more popular lately. Arteriography is able to identify the precise location of the persistent bleeding while open surgery can be limited by inability to localize and ligate the bleeding vessel [22]. Arteriography with selective embolization of the epigastric arteries has been proved to be a primary therapeutic option for hematomas related to low molecular weight heparin by some authors [23, 24]. Additionally, some reports suggest that pulsed US therapy could achieve resolution of the hematoma much earlier than with the conservative management [25]. It is recommended though for organized and nonrecent hematomas, due to the risk of new bleeding. Unfortunately, these newer methods are not available in all medical facilities.

Regarding prognosis, prompt diagnosis leads usually to a good outcome and resolution without sequelae is the rule, as it was in our case [26]. In some cases, SRSH can lead to serious complications including infection, acute renal failure, myocardial infarction, hypovolemic shock, myonecrosis, and small bowel infarction [27, 28]. However, SRSH is rarely fatal. The overall mortality in patients with SRSH has been reported to be 4%. The mortality is higher (25%) in patients undergoing anticoagulation therapy, whereas mortality rates in iatrogenic RSH and pregnant patients are 18% and 13%, respectively [29]. The high mortality is related to the larger hematomas as well as the increased age and significant comorbidities of these patients. Early diagnosis likely reduces the mortality rate, but no studies to date are available to demonstrate this. The morbidity of SRSH is primarily the result of incorrect diagnosis leading to unnecessary exploratory laparotomy, delay in cessation of anticoagulant therapy, or delay in fluid resuscitation and blood transfusion.

In conclusion, SRSH in elderly is a medical emergency, where the atypical clinical presentation of this entity can delay diagnosis and proper management. The early diagnosis of SRSH is the most important factor of low mortality in the elderly, preventing the unnecessary surgical intervention and determining the success of conservative treatment. Conservative management remains the first therapeutic choice for the elderly.
